# Occurence of Ochratoxin A and Biogenic Amines in Croatian Commercial Red Wines

**DOI:** 10.3390/foods8080348

**Published:** 2019-08-15

**Authors:** Paula Žurga, Nada Vahčić, Igor Pasković, Mara Banović, Mladenka Malenica Staver

**Affiliations:** 1 Teaching Institute of Public Health of Primorsko-Goranska County, Krešimirova 52a, HR-51000 Rijeka, Croatia; 2 Faculty of Food Technology and Biotechnology, University of Zagreb, Pierottijeva 6, HR-10000 Zagreb, Croatia; 3 Institute of Agriculture and Tourism, Karla Huguesa 8, HR-52440 Poreč, Croatia; 4 Department of Biotechnology, University of Rijeka, Radmile Matejčić 2, HR-51000 Rijeka, Croatia

**Keywords:** Croatian red wines, HPLC, ochratoxin A, biogenic amines

## Abstract

Food safety is one of the main concerns in the world and in wine it depends mostly on metabolites of microbial origin. The aim of this study was to investigate the occurrence of natural contaminants, ochratoxin A and biogenic amines (cadaverine, histamine, putrescine and tyramine), in Croatian commercial red wines originating from different Croatian wine-making regions. Ochratoxin A was detected in 92.8% of samples, however its concentrations in all samples were more than 10-fold lower than the limit set by the European Union (2 µg/kg), marking these wines as safe for consumption. The frequency of occurrence and measured concentrations of ochratoxin A were higher in wines produced in southern regions with highest values obtained in wines from southern Dalmatian islands. All samples were contaminated with cadaverine and putrescine, while 88.2% and 82.7% were contaminated with histamine and tyramine, respectively. Histamine concentrations ranged from below the limit of detection to 8.5 mg/L. Higher histamine concentrations were measured in wines with higher pH values which coincided with southern geographic origin. These results reinforce the need for routine detection and quantification of biogenic amines in Croatian wines to achieve better control of vinification and minimize their formation.

## 1. Introduction

Ochratoxin A (OTA) is a widely distributed mycotoxin produced as a secondary metabolite by few filamentous fungi from *Aspergillus* and *Penicillium* genera. OTA occurs in cereals, beans, nuts, spices, dried fruits, coffee, milk, beer, grape and grape derived products [1]. Wine is, after cereals, the major source of daily OTA intake in Europe [2]. Concentration of OTA in wine depends on climate (especially on temperature and relative humidity in the month before harvest), grape-growing conditions (including use of fungicides), percentage of damaged berries before maceration and type of maceration [3,4]. It was found that its concentration in wine is dependent on the latitude of the production region: Wines produced from grapes grown in lower latitudes are more frequent in occurrence and greater in concentration of OTA contamination [3]. 

OTA has been associated with nephrotoxic, neurotoxic, hepatotoxic, teratogenic, immunotoxic and cancerogenic effects [5]. International Agency for Research on Cancer (IARC) has classified it as a possible human carcinogen (Group 2B) [6]. Since the presence of OTA in blood of healthy individuals confirms continuous and widespread exposure [7], European Food Safety Agency (EFSA) has set the OTA Tolerable Weekly Intake (TWI) to 120 ng OTA/kg body weight [8]. In the European Union (EU), maximum allowed concentration of OTA in wine is 2 µg/kg [9].

Biogenic amines (BA) are nitrogenous bases of low molecular mass produced mainly by microbial decarboxylation of corresponding amino acids. They are frequently found in fermented foods and beverages, wine being one of them. The most common BA found in wine are putrescine (typically the most abundant), followed by histamine, tyramine, cadaverine, spermine and spermidine and the reported ranges of total BA span from traces to 130 mg/L [10,11]. BA are produced during both alcoholic fermentation (due to yeast metabolism) and malolactic fermentation (due to proliferation of lactic acid bacteria) [11]. Their concentrations in wine depend on numerous factors, such as the climate and soil of the wine-making region, grape ripeness and the amino acid content of grapes (depending also on use of nitrogen fertilizers) as well as on wine-making and storage conditions [12,13,14]. Poor sanitary conditions of the grapes and possible microbial contamination during winery operations may also relate with the higher BA content in wine [15,16]. BA are considered essential for many physiological functions, including control of gastric acid secretion, brain activity and body temperature regulation [17]. However, ingestion of large amounts of BA as well as inhibition of their normal catabolic routes may lead to onset of symptoms that include migraine, nausea, hyper- or hypotension, cardiac palpitations, skin irritation and respiratory distress [18]. The ingestion of BA through wine is of great concern because the interaction of BA and ethanol seems to be synergistic and enhance their toxic effects [18]. Histamine and tyramine are considered the most toxic BA and are particularly relevant to food safety [19]. On the other hand, polyamines such as putrescine and cadaverine, although indispensable for living cells and without toxic actions, can inhibit enzymes involved in the degradation of histamine and tyramine and reinforce their toxicity [11].

The wide range of BA found in wine is a source of concern in many countries and efforts have been invested into better understanding and control of BA formation during the vinification process [20,21,22]. However, the EU has set no maximum allowed concentration neither for histamine nor for tyramine in wine due to the lack of reliable pharmacological data on critical BA concentrations that cause toxic effects in different populations and lack of knowledge about other wine ingredients that might influence the release of endogenous histamine [11,23]. 

Croatia, like many other Mediterranean countries, has a long-standing wine-making tradition. The Croatian market is known as a market of a large number of monovarietal wines, due to a large number of small manufacturers which produce small quantities of wine, often made not only from well renowned international, but also from autochthonous as well as rare grape varieties [24]. However, according to our knowledge, reports on OTA and BA concentrations in Croatian commercial wines are scarce. Domijan and Peraica [25] analyzed OTA in seven white and seven red Croatian wine samples while Flajs et al. [26] analyzed 10 red wines for OTA contamination. Recently, Mitar et al. [27] analyzed 11 BA in 21 white and 27 red wine samples from Croatia aiming to compare BA concentrations from red and wine wines as well as to compare BA concentration in wines from different geographic origin. Given the known toxic effects of both BA and OTA, and given the lack of more comprehensive information about their concentration in Croatian red wines, the aim of this study was to investigate the occurrence of these natural contaminants in Croatian commercial red wines. Moreover, it has been recently suggested, due to observed great increase of OTA in wine on a decadal scale, that climate changes may have profound influence on OTA concentration which necessitates continuous OTA monitoring in wine [28]. Therefore, this info will provide a valuable insight into the current status of natural contaminants in Croatian wines and into critical properties of these wines that are favorable for occurrence of investigated compounds. 

## 2. Materials and Methods

### 2.1. Chemicals

Acetonitrile (AcN) (HPLC grade), perchloric and acetic acid as well as acetone were from Merck (Darmstadt, Germany). Potassium dyhydrogen phosphate, phosphoric acid, sodium hydrogen carbonate, sodium hydroxide, sodium chloride and polyethylene glycol 8000 were purchased from Sigma-Aldrich (St. Louis, MO, USA). Ammonia solution (25%) was from Kemika (Zagreb, Croatia).

Standards of malic acid, lactic acid, ochratoxin A (10 µg/mL in acetonitrile) as well as BA: Putrescine, cadaverine, histamine, tyramine, derivatizating agent dansyl chloride and internal standard 1.7-diaminoheptane were from Sigma-Aldrich (St. Louis, MO, USA). 

OTA standard solutions for calibration purpose were prepared by dissolving aliquots of stock solution in the mobile phase. Stock standards of each BA and the internal standard (1000 mg/L) were prepared with deionised water (Siemens Water Technologies Corp, Warrendale, PA, USA). 

### 2.2. Samples

A total of 110 widely consumed, Croatian commercial red wines in glass bottles (0.75 L), all labeled with protected designation of origin (PDO), were collected directly from the local markets and wine shops. Samples were produced from seven grape varieties, three autochthonous (Babić, Plavac mali and Teran) and four introduced (Blaufränkisch, Cabernet Sauvignon, Merlot and Pinot noir), originating from three grape growing regions: Dalmatia, Istria and Eastern Continental Croatia. All the wines were produced over the years 2011–2015, stored in darkness at 12–15 °C and analyzed immediately after opening. The detailed description of samples is presented in Table 1.

### 2.3. Basic Parameters

Total sulphur dioxide, pH and ethanol content were determined by the EU recommended methods for wine sector (Regulation No. 2676/1990) [29]. Malic and lactic acid were determined by HPLC method as described by Zheng et al. [30].

### 2.4. OTA Analyses

OTA was analyzed by HPLC with fluorescence detection after immunoaffinity column (IAC) clean up as described by Visconti et al. [31]. Immunoaffinity OchraTest columns were purchased from Vicam, Waters (Milford, MA, USA) and purification was carried out according to the manufacturer’s instructions. The analytical column was Lichrospher 100 RP-18 (250 mm × 4 mm, 5 µm) with pre-column Lichrospher 100 (4 × 4 mm, 5 µm), both supplied by Agilent Technologies (Santa Clara, CA, USA). The mobile phase consisted of AcN:H_2_O:acetic acid (99:99:2), kept at a constant flow rate of 1 mL/min. Fluorescence detection was set at 333 nm for excitation and 460 nm for emission. Injection volume was 100 µL.

### 2.5. BA Analyses

BA (putrescine, histamine, tyramine and cadaverine) were determined by HPLC with UV/Vis detection and pre-column derivatization with dansyl chloride as previously described [32]. The excess of dansyl chloride was removed after derivatization by adding 100 µL NH_4_OH 25% (v/v). The analytical column was Lichrospher 100 RP-18 (250 mm × 4 mm, 5 µm) with pre-column Lichrospher 100 (4 × 4 mm, 5 µm), both supplied by Agilent Technologies (Santa Clara, CA, USA). Gradient elution with two solvents, (A) H_2_O and (B) AcN, was used. The chromatographic conditions were as follows: 50% B 0–3 min; 5–90% B 3–23 min; 90% B 23–26 min; 90–50% B 26–30 min; 50% B 30–40 min, delivered at constant flow rate of 1.2 mL/min. Temperature was held at 25 °C. UV/Vis detection was set at 254 nm.

### 2.6. Validation of Applied Technology

The limit of detection (LOD) was obtained by serial dilution of stock standard solutions of each analyte until the peak heights were three times the standard deviations of the blank signal calculated over ten injections of the blank. Recovery was determined by spiking blank wine sample with different aliquots of stock standard solutions to obtain final concentrations of 0.02, 0.05 and 0.1 µg/L of OTA, and 1, 5 and 10 mg/L of investigated BA in wine. Each recovery experiment was performed in triplicate. The relative standard deviation (RSD) was used to evaluate the precision of the method. The within-day precision was tested by analyzing spiked wines six times within the same day. The results of validation experiments are presented in Table 2.

### 2.7. Statistical Analysis

All measurements were conducted in triplicate. Pearson correlation coefficients were calculated to assess relationship between parameters. For statistical purposes, values below LOD were replaced with LOD/2. Statistica 10.0 software (Stat-Soft, Tulsa, OK, USA) was used for statistical procedures. The probability of *p* < 0.05 was considered statistically significant.

## 3. Results and Discussion

A wide range of Croatian monovarietal red wines was analyzed for OTA and BA contamination. The sample represented geographically diverse sources, as it included both wines from continental (Eastern Continental Croatia) and maritime regions of Croatia (Istria and Dalmatia), characterized by distinguished climatic conditions. Eastern Continental Croatia has a continental climate with hot summers and cold winters and enough rainfall to make it a major agricultural area. The coastal line has Mediterranean climate, however, Istria is influenced also by the Alpes and has hot and humid summers and mild winters, while Dalmatia has hot and dry summers and mild winters. The investigated wines can further be traced by their protected designation of origin (PDO) to the wine-making sub-regions: Slavonia and Croatian Podunavlje within Eastern Continental Croatia; Croatian Istria; Northern Dalmatia, Dalmatian Inland, Central and Southern Dalmatia and Dingač within Dalmatia. Due to greater prevalence of red varieties in maritime regions [33] more wines in this research were sourced from Istria and, especially, Dalmatia.

### 3.1. Ochratoxin A

OTA concentrations in analyzed wines ranged from below LOD to 0.163 µg/L, while the average and median values were 0.040 and 0.026 µg/L, respectively, with only 9% of wines having OTA concentration higher than 0.1 µg/L (Table 3 and Table 4). Since this is the first comprehensive report of OTA in Croatian wines, the results are extensively compared with those from different European and Mediterranean countries (Table 5). However, comparisons with results of other authors have to be done with caution, since methodologies of analytical procedures as well as of reporting of results differ notably. In an attempt to present more uniform results from previous studies, Table 5 contains the results obtained by methods using mostly IAC clean-up of samples followed by HPLC with fluorescence detection, although results obtained by different sample clean-up procedures or those with no clean-up are also presented (direct injection, C18-SPE) resulting mostly in higher LOD or limit of quantitation (LOQ) values. In addition, Remiro et al. [34] argued that the obtained results were much better represented by median than by average values, so both values were included, where possible. 

The range of ochratoxin A concentrations in wines in our research was somewhat larger than that previously reported for Croatian wines [25,26] (Table 5), however, the average and median values showed only a small increase in spite of the substantial span of investigated years (2002 in previous studies-2015 in present study), contrary to the observations of De Jesus et al. [28] who found nearly two orders of magnitude increase on a decadal scale, although any conclusion based on these findings must be drawn with caution due to much smaller number of red wine samples in previous Croatian studies (seven and ten samples, respectively). The most significant OTA producing species are *Penicillium verrucosum*, *Aspergillus ochraceus*, *Aspergillus niger* and *Aspergillus carbonarius* [35], the last one being the main producer [36]. Southern Europe is more favorable for growth of ochratoxigenic *Aspergillus* than *Penicillium* species, since black aspergilli are very resistant to sunshine exposure and to a hot and dry climate [37,38] which gives them competitive advantage in comparison with other fungi. However, climate change, especially the rise of average temperatures, will probably lead to substantial changes in OTA production on a decadal scale. Different OTA producing species differ in optimal growth and optimal OTA producing temperatures. Higher OTA concentrations were measured in grapes at 30 °C (upper limit of optimal growth temperature span for *Aspergillus carbonarius* and lower limit of optimal growth temperature span for *Aspergillus niger*) than in grapes at 20 °C [38,39]. With climate change, it is likely that *Aspergillus niger* will become prevalent over *Aspergillus carbonarius* since it is better adapted to extreme high temperatures and dry conditions [40] but produces OTA less frequently [41,42]. Therefore, the rise of temperatures in moderate climates will most probably lead to a rise of OTA concentrations, while a rise of temperatures to extreme values such as in the Southern regions of Europe will probably lead to decline in OTA concentrations. However, other mycotoxins might become more present in wine, such as aflatoxin, since it is produced by more thermoresistant fungi [38]. Therefore, small increase of OTA concentration in Croatian wines in comparison with previous reports supports these assumptions, since most of the samples originate from regions with an already hot climate, where further increase of temperatures will probably not lead to further substantial increase in OTA concentrations. Future studies are needed to investigate this proposed trend.

**Figure 1:**
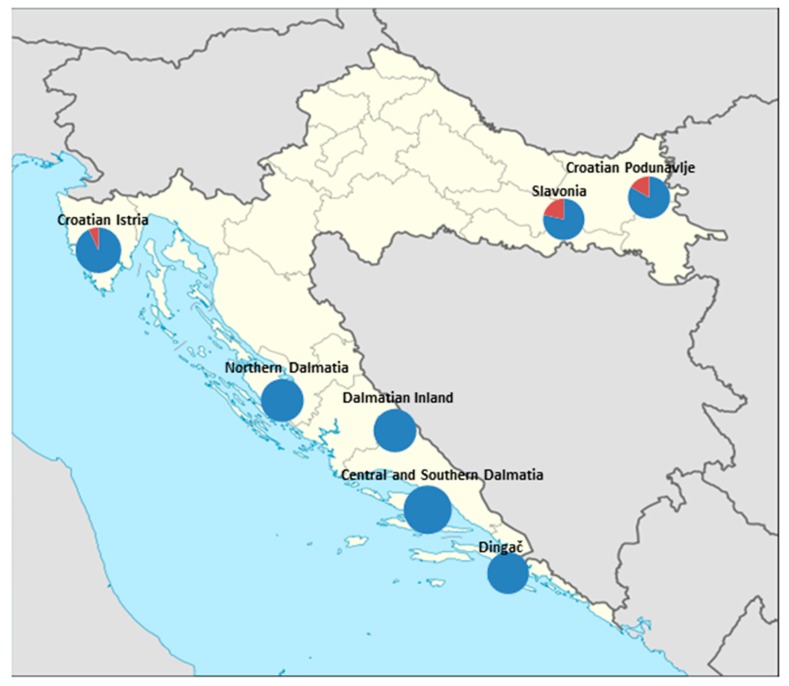
Incidence of contaminated samples according to geographic origin (contaminated-blue; non-contaminated-red).

Compared to numerous reports on internationally sourced wines (Table 5), Croatian wines were seemingly more comparable to wines from Central European countries [43,44,45] although Croatian South (Dalmatia), from which the majority of our samples originated, has typical Csa climate (warm temperature-summer dry-hot summer) of Koppen-Geiger climate classification [46] which corresponds to climate characteristics of Southern European and Mediterranean countries [47,48,49,50,51,52,53,54,55] (Table 5) with higher reported concentrations of OTA. Owing to the low limit of detection, the incidence of contamination occurrence was high (92.8%) but comparable to those previously reported for Croatian wines [25,26,34]. According to geographic origin of investigated wines, there was a gradual southwards increase in OTA concentrations with the highest individual concentrations measured in samples produced in southernmost areas of investigated regions (0.163 µg/L in wine from island Vis and 0.141 µg/L in wine from island Hvar). In addition, the frequency of occurrence of OTA contamination increased southwards, as all Dalmatian wines were contaminated (Figure 1), as well as the prevalence of high level of contamination vs. low level of contamination (Table 4). 

These results strongly support previous findings, as reviewed by Blesa et al. [57], where wines from southern European regions contained higher OTA concentrations, although a more recent study [34] has questioned this trend. Higher concentrations of OTA in southern regions, not only on international scale, but also on a within-country scale, were also observed by Labrinea et al. [49] and Brera et al. [51]. When comparing the results from different PDOs, the highest values of OTA were measured along the Dalmatian coast, while values measured in wines originating from geographically close and the climatologically very similar Dalmatian Inland were comparable to those measured in continental wines. Although the average daily temperatures and the total rainfall amount are similar in those regions, the Dalmatian Inland does not enjoy the temperature regulation provided by the sea, and is characterized by sharp differences between day-night temperatures. It can be hypothesized that lower night temperatures were responsible for lower measured concentrations of OTA in wines originating from Dalmatian Inland. In fact, Clouvel et al. [58] have suggested that minimum air temperature of 15 °C during the berry contamination period might correspond to a lower limit below which the fungal growth is restricted. In addition, higher concentration of OTA in coastal wines can be explained by higher air humidity provided by the sea, as previously observed [53]. It is interesting to note that highest OTA concentrations (highest average, highest maximum) (data not shown) were measured in wines from the climatologically unfavorable year of 2014. In addition, all the wines from 2014 were contaminated with OTA. Year 2014 was the most humid among studied years (+32% on annual level) with temperatures slightly lower than average or without deviation from the average. The greatest increase of rainfall amount occurred in July–September period which probably favored the growth of the ochratoxigenic species [58]. 

It is difficult to make any conclusion about the variety-dependent level of contamination since each variety (with exception of Merlot and Cabernet Sauvignon) originated from one region and, therefore, different varieties were grown under different climatic conditions. However, the frequency of occurrence of OTA contamination pointed towards varietal dependence. Blaufränkisch and Merlot were the only wines with non-contaminated samples (22.2%). Considering the dominant effect of climate on OTA contamination, Merlot wines were compared with Cabernet Sauvignon wines (all samples contaminated) since both of these were the only varieties with samples originating from all three studied regions. The result points to variety-dependent occurrence of OTA contamination and suggests the influence of employed viticultural practices. This could be particularly true when considering the time of harvest [59] since Merlot grapes are known to ripen earlier than Cabernet Sauvignon, which reduces the risk of optimal growth of OTA producing strains at higher temperatures. In addition, frequency of occurrence of OTA contamination in Blaufränkisch wines was compared to that of Pinot noir wines (all wines contaminated) since both grape varieties were grown in the same region (Eastern Continental Croatia). This difference could be explained with the fact that Pinot noir has much thinner skin in comparison to Blaufränkisch and is highly susceptible to pathogenic fungi, like *Botrytis cinerea*, which damage the berry and create conditions favorable for colonization of ochratoxigenic fungi [59].

### 3.2. Biogenic Amines

All studied wines were contaminated with putrescine and cadaverine, 88.2% with histamine and 82.7% with tyramine. Cabernet Sauvignon wines seemed to be the most and Merlot wines the least susceptible to BA contamination (Table 6). 

In all investigated wines, putrescine was the most abundant, followed by histamine, tyramine and cadaverine (Table 6) which is in agreement with previous findings [23]. The ranges of reported results for individual BA were mostly consistent with recent results reported for Italian wines [60]. However, Mitar et al. [27] found much lower BA values in Croatian wines. Whether these differences are real features of investigated wines (the study of Mitar et al. included also wines from Western Continental Croatia which were not part of our study), or are consequence of methodologic discrepancies, must remain open for the time being. However, in previous reports, as reviewed by Anzin-Azpilicueta et al. [11], BA concentrations varied greatly, with ranges from below detection limit to the following amounts: 55 mg/L for putrescine, 25 mg/L for histamine, 28 mg/L for tyramine and 14 mg/L for cadaverine. Konakovsky et al. [23] reported even higher values for putrescine (122 mg/L) and histamine (27 mg/L) measured in Austrian high-quality red wines. This variability is expected, since BA concentrations depend on various factors, such as the quality of raw material (depending on climate and soil characteristics of wine-making region), aminogenic capabilities of present microbial strains, the vine-growing conditions (including irrigation and use of nitrogen fertilizers), wine-making techniques (including type of maceration and fermentation conditions) and storage conditions [17]. Due to great number of influencing factors, we observed high within-group (variety) variability which is in agreement with the findings of Konakovsky et al. [23] who, for the same reason, did not find statistically significant differences between wines produced from seven red varieties. 

When observing geographic origin of the samples, wines with highest average histamine concentrations had higher pH values: PDO Dingač (4.2 mg/L, pH = 3.74); PDO Central and Southern Dalmatia (2.4 mg/L, pH = 3.53); PDO Northern Dalmatia (4.3 mg/L, pH = 3.50), while wines with lower pH values had lowest average histamine concentrations: PDO Dalmatian Inland (0.9 mg/L, pH = 3.22); PDO Croatian Podunavlje (1.1 mg/L, pH = 3.38). Average pH values increased southwards which is consistent with the fact that grapes which grow in warmer climate have less acids in comparison with grapes from colder climate. Higher pH values (above 3.5) are connected with higher microbial surviving abilities, while lower pH values (below 3.3) may cause difficult malolactic fermentation [61]. Data of malic and lactic acid concentrations show that all the wines had gone through partial or complete malolactic fermentation: The malic acid concentrations of investigated wines ranged from not detected to 1927 mg/L, while lactic acid concentrations ranged from 519 to 3982 mg/L. Higher pH values (demonstrated with positive correlation of lactic acid and negative correlation of malic acid with pH values, as shown in Table 7) in wine that had undergone malolactic fermentation were expected, since microbial malic acid degradation and formation of lactic acid is accompanied by slight increase in pH values. Concomitant synthesis of BA was suggested as microbial strategy to survive acidic environment or to provide alternative metabolic energy [62]. Positive correlation between pH and BA concentrations was previously reported [12,60,61]. Consistent with our findings, Landete et al. [12] measured higher histamine concentrations in wines with pH values above 3.5 while the influence of pH was less pronounced for tyramine and not observed for other BA. In our study, statistically significant correlation coefficients between pH values and histamine and tyramine concentrations support these findings (*r* = 0.304, *p* < 0.05; *r* = 0.300, *p* < 0.05, respectively). In addition, malic acid was negatively correlated with histamine (*r* = −0.328, *p* < 0.05), lactic acid was positively correlated with tyramine (*r* = 0.302, *p* < 0.05), while both histamine and tyramine were highly correlated (*r* = 0.605, *p* < 0.05) indicating their common origin in malolactic fermentation (Table 7). Although some authors found positive correlation between BA concentration and ethanol, considering it as enhanced risk for some consumers [61], we observed no connection between BA concentrations and neither ethanol content nor SO_2_ concentrations. Considering changes of biogenic amines concentrations during conservation, no correlations were observed between age of wine and biogenic amines in investigated wines (Table 7), contrary to the several previous reports where histamine was found either to increase in the first six months and then decrease afterwards [12], increase in the first 105 days [63] or to decrease [64] during storage. However, these studies were conducted over a relatively short period of time (maximum of 12 months) so studies evaluating longer periods of conservation, especially important for high quality wines, should be performed. 

## 4. Conclusions

Monitoring of OTA concentrations in wine is of great importance due to high incidence of its occurrence in wine as well as because of its mutagenic and toxic potential. In addition, the concerns regarding the impact of climate change require continuing efforts in establishing possible trends of OTA concentrations in wine. Croatian wines from southern regions and from climatologically unfavorable vintage contained higher concentrations of OTA and frequency of occurrence of OTA contamination was not only geographically-dependent, but also variety-dependent. Comparison with previous Croatian studies showed only a small increase of OTA in wine on a decadal scale. In all investigated wines, OTA concentrations were more than 10-fold lower than its maximum allowed concentration, 2 µg/kg, set by the EU, marking these wines as safe for consumption. 

Putrescine and cadaverine occurred in all samples, putrescine being the most and cadaverine the least abundant. Histamine and tyramine were not detected in all samples showing that production of wines devoid of these toxicologically important BA is technologically possible. The concentrations of histamine in studied wines were pH-dependent. In spite of potentially toxic effects on sensitive individuals, the EU has set no maximum allowed concentration neither for histamine, nor for the other pharmacologically active amine, tyramine. Switzerland was the only country that temporarily set the legal limit for histamine (10 mg/L) but has withdrawn it when adjusting regulations with current legislation of the EU. Applying this threshold would mark all investigated wines as legally safe. However, possible reactions to patients with histamine intolerance could not be ruled out having in mind the synergistic effect of different BA present in wine and the fact that other food potentially contaminated with BA can be eaten at one meal thus increasing the content of ingested biologically active BA. Therefore, continuous monitoring of BA in wine is necessary and, in absence of regulatory limit, BA labeling would be most useful measure of caution for sensitive individuals.

## Figures and Tables

**Table 1:** Classification of wine samples according to variety, geographic origin and vintage.

Variety	Region	Protected Designation of Origin (PDO)	Vintage (No. of Samples)
Babić	Dalmatia	Northern Dalmatia	2011(1); 2012(1); 2013(2); 2014(1); 2015(1)
Blaufränkisch	Eastern Continental Croatia	Croatian Podunavlje	2012(1); 2013(1); 2015(1)
Blaufränkisch	Eastern Continental Croatia	Slavonia	2012(3); 2013(1); 2015(2)
Cabernet Sauvignon	Eastern Continental Croatia	Croatian Podunavlje	2013(2); 2014(1); 2015(2)
Cabernet Sauvignon	Eastern Continental Croatia	Slavonia	2014(2); 2015(1)
Cabernet Sauvignon	Istria	Croatian Istria	2011(2); 2012(1); 2013(3); 2014(1); 2015(3)
Cabernet Sauvignon	Dalmatia	Northern Dalmatia	2012(1)
Cabernet Sauvignon	Dalmatia	Dalmatian Inland	2013(1)
Merlot	Eastern Continental Croatia	Croatian Podunavlje	2011(1); 2012(1); 2013(1); 2014(1); 2015(2)
Merlot	Eastern Continental Croatia	Slavonia	2012(1); 2015(2)
Merlot	Istria	Croatian Istria	2012(1); 2013(5); 2014(1); 2015(2)
Merlot	Dalmatia	Northern Dalmatia	2013(1)
Merlot	Dalmatia	Dalmatian Inland	2013(2); 2014(1); 2015(2)
Merlot	Dalmatia	Central and Southern Dalmatia	2011(1); 2014(1); 2015(1)
Pinot noir	Eastern Continental Croatia	Croatian Podunavlje	2012(2); 2015(2)
Pinot noir	Eastern Continental Croatia	Slavonia	2012(1); 2013(1)
Plavac mali	Dalmatia	Central and Southern Dalmatia	2013(7); 2014(9); 2015(11)
Plavac mali	Dalmatia	Dingač	2013(3); 2015(2)
Teran	Istria	Croatian Istria	2011(1); 2012(2); 2013(2); 2014(1); 2015(4)

**Table 2:** The results of methods’ validation experiments.

	Limit of Detection	Spiked	Recovery (%)	Within-Day Precision * RSD (%)
OTA (µg/L)		0.02	88.2	9.8
	0.006	0.05	91.1	8.2
		0.1	93.6	6.9
Putrescine (mg/L)		1	90.1	8.8
	0.1	5	92.8	6.9
		10	93.3	6.8
Histamine (mg/L)		1	92.1	7.5
	0.2	5	92.8	7.2
		10	96.5	5.9
Tyramine (mg/L)		1	92.6	8.7
	0.2	5	95.2	8.3
		10	101.1	5.8
Cadaverine (mg/L)		1	89.6	9.2
	0.2	5	92.2	7.5
		10	94.9	6.9

OTA—Ochratoxin A; RSD—relative standard deviation. * Within-day precision was tested by analyzing spiked wines six times within the same day.

**Table 3:** Concentrations of ochratoxin A (µg/L) according to geographic origin.

Region (No of Samples)	Protected Designation of Origin (PDO)	Average	Median	Range
All regions (110)		0.040	0.026	0.003–0.163
Dalmatia (49)	Dalmatian Inland	0.020	0.018	0.007–0.036
	Dingač	0.095	0.088	0.062–0.124
	Northern Dalmatia	0.065	0.057	0.025–0.106
	Central and Southern Dalmatia	0.061	0.051	0.009–0.163
Istria (29)	Croatian Istria	0.020	0.016	0.003–0.068
Eastern Continental Croatia (32)	Croatian Podunavlje	0.028	0.020	0.003–0.079
	Slavonia	0.023	0.017	0.003–0.060

**Table 4:** Prevalence of measured concentrations of OTA according to geographic region.

Region (No. of Samples)	Protected Designation of Origin (PDO)	Number of Wines in the Concentration Range			
		<0.02 µg/L *	0.02–0.05 µg/L	0.05–0.1 µg/L	>0.1 µg/L
All regions (110)		45 (41%)	32 (29%)	23 (21%)	10 (9%)
Dalmatia (49)	Dalmatian Inland	3 (50%)	3 (50%)	0 (0%)	0 (0%)
	Dingač	0 (0%)	0 (0%)	3 (60%)	2 (40%)
	Northern Dalmatia	0 (0%)	2 (33%)	3 (50%)	1 (17%)
	Central and Southern Dalmatia	7 (22%)	8 (25%)	10 (31%)	7 (22%)
Istria (29)	Croatian Istria	18 (62%)	9 (31%)	2 (7%)	0 (0%)
Eastern Continental Croatia (32)	Croatian Podunavlje	9 (50%)	6 (33%)	3 (16%)	0 (0%)
	Slavonia	8 (57%)	4 (29%)	2 (14%)	0 (%)

* This category also includes wines with ochratoxin A concentrations below limit of detection (LOD).

**Table 5:** Ochratoxin A incidence of wine contamination and concentrations measured in wine from previous studies.

Country	No. of Samples	Vintages	Contaminated Samples (%)	LOD (µg/L)	Range (µg/L)	Mean (µg/L)	Median (µg/L)	Analytical Procedure	Reference
Slovakia	39	2005	31	0.011	<LOD–0.463	-	-	IAC HPLC FLD	[43]
Moravia	46	2003–2009	11	0.0003	<LOD–0.071	-	-	IAC UPLC FLD	[44]
Hungary	65	2000–2002	97.7	0.01	0.01–0.533	0.110	-	Immunoassay	[45]
Croatia	14	2002–2003	79	0.010	<LOD–0.047	0.022	0.022	IAC HPLC FLD	[25]
Croatia	10	2007	80	0.005	<LOD–0.021	0.015	0.015	IAC HPLC FLD	[26]
Croatia	-	2004–2008	100	0.00032	0.00036–0.061	0.0188	0.00765	IAC HPLC FLD	[34]
France	49	2002–2005	96	0.01	<LOD–1.218	-	-	IAC HPLC FLD	[47]
France	-	2006–2009	91.7	0.00032	<LOD–0.088	0.0336	0.00356	IAC HPLC FLD	[34]
Portugal	60	-	20	1 (LOQ)	<LOQ–2.4	-	-	Direct injection HPLC FLD	[48]
Spain	100	1995–2008	57	0.002	<LOD–0.179	0.035	-	IAC HPLC FLD	[56]
Spain	188	2002–2005	99	0.01	<LOD–4.63	0.65	0.19	IAC HPLC FLD	[47]
Spain	-	2006–2009	100	0.00032	0.00105–0.104	0.0372	0.0251	IAC HPLC FLD	[34]
Greece	150	1999–2006	69.4	0.01	<LOD–2.00	0.26	0.10	IAC HPLC FLD	[49]
Greece	-	2003–2007	100	0.00032	0.00435–0.212	0.0594	0.0317	IAC HPLC FLD	[34]
Greece	60	2007–2009	86.7	0.01	<LOD–2.52	0.38	0.10	IAC HPLC FLD	[50]
Italy	1166	1988–2004	64.32	0.01	<LOD–7.5	0.28	0.07	IAC HPLC FLD	[51]
Italy	1206	2000–2007	68	0.0072	<LOD–2.63	0.116	-	IAC HPLC FLD	[52]
Italy	-	2005–2008	100	0.00032	0.00518–0.286	0.0539	0.020	IAC HPLC FLD	[34]
Turkey	95	-	86	0.006	<LOD–0.815	-	-	IAC HPLC FLD	[53]
Turkey	47	1999–2001	100	0.01	0.02–2.23	0.419	-	SPE clean up HPLC FLD	[54]
Turkey	25	1987–2005	100	0.052	0.25–7.96	2.55	-	Direct injection HPLC FLD	[55]
Turkey	-	2003–2007	100	0.00032	0.00291–0.101	0.0311	0.0205	IAC HPLC FLD	[34]

LOD—limit of detection; LOQ—limit of quantitation; IAC—immunoaffinity column clean up; SPE—solid phase extraction; FLD—fluorescence detection.

**Table 6:** Concentrations of biogenic amines (mg/L) according to grape variety.

		Babić (*n* = 6)	Blaufränk. (*n* = 9)	Cabernet Sauvignon (*n* = 20)	Merlot (*n* = 27)	Pinot Noir (*n* = 6)	Plavac Mali (*n* = 32)	Teran (*n* = 10)
Putrescine	Average	6.5	5.5	6.5	5.2	3.3	6.0	4.7
	Min.–max.	3.5–9.1	3.3–9.6	3.4–14.1	1.0–13.4	2.0–5.8	1.5–14.0	1.8–9.2
	Contaminated samples (%)	100	100	100	100	100	100	100
Histamine	Average	3.8	2.0	2.1	1.0	0.9	3.0	2.2
	Min.–max.	0.9–7.1	0.4–4.6	0.1–8.5	0.2–3.8	0.1–3.3	0.1–7.1	0.1–4.4
	Contaminated samples (%)	100	100	100	74.1	100	87.5	80
Tyramine	Average	1.5	2.4	1.5	0.7	0.5	1.9	1.5
	Min.–max.	0.1–3.5	0.1–8.4	0.1–5.9	0.1–3.5	0.1–0.9	0.1–8.0	0.1–3.6
	Contaminated samples (%)	83.3	88.9	90	81.5	83.3	78.1	80
Cadaverine	Average	0.8	0.9	0.9	0.8	0.6	1.0	0.9
	Min.–max.	0.2–1.3	0.2–2.0	0.3–2.2	0.1–2.7	0.2–1.0	0.3–3.0	0.3–3.0
	Contaminated samples (%)	100	100	100	100	100	100	100

**Table 7:** Correlations between vintage, basic parameters and concentrations of biogenic amines in investigated wines.

	Vintage	Ethanol	SO_2_	pH	MA	LA	PUT	CAD	HIS	TYR
Vintage	1.000									
Ethanol	−0.052	1.000								
SO_2_	0.029	−0.212	1.000							
pH	0.080	0.126	−0.101	1.000						
MA	−0.045	0.024	0.201	**−0.312**	1.000					
LA	0.053	**−0.357**	−0.148	**0.430**	**−0.315**	1.000				
PUT	−0.002	−0.055	−0.062	−0.151	0.075	−0.017	1.000			
CAD	0.247	0.038	0.102	0.037	0.100	0.000	−0.038	1.000		
HIS	0.082	−0.189	−0.105	**0.304**	**−0.328**	0.156	**0.362**	0.059	1.000	
TYR	−0.004	−0.121	−0.022	**0.300**	−0.140	**0.302**	**0.410**	−0.004	**0.605**	1.000

MA—malic acid; LA—lactic acid; PUT—putrescine; CAD—cadaverine; HIS—histamine; TYR—tyramine. The statistically significant correlation coefficients are marked in bold (*p* < 0.05).
